# Construction of Computer English Corpus Assisted by Internet of Things Information Perception and Interaction Technology

**DOI:** 10.1155/2022/6803802

**Published:** 2022-10-11

**Authors:** Miao Tian

**Affiliations:** School of Foreign Languages, Weinan Normal University, Weinan 714099, Shaanxi, China

## Abstract

Aiming at the situation that multiple sensing devices in the sensing layer sense the same sensing quantity, multiple redundant measurement data will be generated. In this paper, an improved Kalman filtering algorithm is used. The algorithm combines with numerical test theory to remove abnormal error data before filtering, thereby reducing the error of filtering result and improving its reliability. The parallel multiprotocol processing mechanism of the gateway is designed, and the IoT gateway has a hierarchical modular processing design for different protocol signals of the heterogeneous perception layer. The web interaction function with good platform versatility is designed to provide a visual interaction interface for the application layer, which is convenient for the application layer terminal to monitor and manage the perception layer equipment. This paper realizes the retrieval and query function of keywords, wrong sentences, and English character errors. This corpus enriches the less existing Madagascar corpus and establishes a separate English character error query database. This paper describes the overall process of corpus from preliminary preparation to establishment and the related use instructions of corpus, makes a statistical analysis of the current corpus data, and puts forward some suggestions for building a computer English corpus according to the analysis results.

## 1. Introduction

Situational awareness is one of the main features of IoT intelligence. The introduction of situational awareness technology enables edge smart devices to perceive situational changes in dynamic environments and to make intelligent decision-making services based on the perceived situation [1]. However, the complexity, dynamics, and distribution of the IoT edge environment have brought great challenges to the application of context awareness technology in the edge environment, such as high-level context awareness, context awareness under incomplete context information, and other research issues [2]. High-level situational awareness supports the identification of complex situations and realizes complex situational awareness mainly by obtaining high-level situational information from low-level situational information. In high-level situational awareness, obtaining complete low-level situational information is the basis for realizing complex situational awareness [3]. However, due to the characteristics of the IoT edge environment and the noise, conflict, unreliable links, and accidental damage in real life, data loss in wireless sensor networks is common, resulting in incomplete low-level contextual information usually obtained. The rule-based context awareness method is the main method to realize complex context awareness in the edge environment [4].

Intelligent interaction technology is an important part in the field of computer science and control scientific research [5]. The current research on intelligent interaction technology mainly focuses on the human-centered, PC-based, and traditional mobile computing “human-computer interaction,” while the research on “object-object interaction” is mainly based on traditional wireless sensor networks. With the advent of the Internet of Things era, the three-dimensional world of human society, information space, and physical world will be fully connected and integrated. The interaction is developing towards an all-round and three-dimensional natural interaction, and the interaction mode also needs to be expanded from a single human-computer interaction mode to a three-dimensional world interaction mode of human, machine, and object [6]. Therefore, studying the key technologies of intelligent interaction in the Internet of Things environment and solving the interaction problems in the Internet of Things environment have important theoretical significance and urgent reality for realizing the comprehensive connection and integration of the three-dimensional world of human society, information space, and physical world [7].

Through the analysis of IoT context-aware services, this paper constructs the IoT edge context-aware service framework, which supports the reasoning of complex scenarios, and introduces a publish/subscribe-based lightweight data communication mechanism to implement services in the edge environment. After analyzing the characteristics of context fusion in the intelligent interactive system under the Internet of Things environment, on the basis of the detailed analysis of the current information fusion level, information fusion structure, and information fusion method, rule management based on rough set theory is introduced. And the generation algorithm and matching algorithm of the rules are described in detail.

The perception layer network is composed of many heterogeneous sensor networks and other device networks. It is characterized by a large number of perception terminals and a very large data traffic. The transmission of huge data traffic will block the network and increase the delay. At the same time, the transmission of these data also requires consuming a lot of energy. In this paper, two methods of data classification and aggregation and data fusion processing are proposed to improve the transmission efficiency of network data in the perception layer and reduce unnecessary data transmission such as redundancy. Among them, data classification and aggregation is a method of reducing the transmission of the same packet header data in the network by aggregating a large number of data packets into a large data packet that aggregates a large amount of useful data information, which improves the transmission ratio of useful information in the network. Data fusion is aimed at the redundant data transmission of multiple sensing layer devices to the same detection object. It performs data fusion processing before the data are transmitted to the IoT gateway. After removing redundant information, the fusion result is uploaded to the Internet of Things. This paper proposes a data fusion method that combines numerical analysis and Kalman filter fusion. It can be seen from simulation verification that this method improves the accuracy and reliability of data fusion compared with the ordinary Kalman filter fusion method.

## 2. Related Work

In the learning-based situational perception method, relevant scholars analyze acceleration sensor data through the backpropagation neural network to perceive human behavioral activities, such as walking, running, sitting, and other basic behaviors [8]. Relevant scholars use deep learning neural networks to analyze mobile sensor data to identify the situational patterns of human activities [9]. In the rule-based situational awareness method, the researchers proposed a situational awareness system based on ontology and rules. The system uses the ontology representation method to model situational information, recognizes complex situational patterns through rule reasoning, and is used for home care scenarios. Relevant scholars have realized situational awareness based on the combination of ontology knowledge and rules, acquired situational knowledge through rule reasoning, and applied them to the centralized environment, distributed environment, and mobile environment, respectively [10].

Related scholars have proposed a rule-based matching strategy to perceive the situation in the smart home environment and provide personalized customized services according to the current situation [11]. In the hybrid context perception method, relevant scholars use neural networks and ontology to build a framework for intelligent IoT systems and obtain low-level contextual information from sensor data through learning-based contextual awareness methods [12]. To achieve complex situation awareness through ontology reasoning, the proposed framework is mainly deployed on cloud servers to provide situation awareness services.

Investigating the research status of IoT situational perception methods shows that learning-based situational perception methods usually use raw perception data to identify human behavior and other situational patterns. Rule-based situational awareness methods are usually combined with ontology modeling methods to identify complex situational patterns through rule-based reasoning. The hybrid situational awareness method mainly integrates the above two methods [13]. The learning-based situational perception method is used to obtain low-level situational information, and based on the low-level situational information, the perception of complex situations is realized through rule reasoning. Existing research studies on perception methods are mainly deployed on cloud servers to provide context-aware services [14].

At present, due to the development of sensing technology, context can be obtained more easily, and the types and quantity of context are also more abundant [15]. The research and utilization of context-aware computing has become very important, and current research mainly focuses on context acquisition, context modeling, context information processing, and building context-aware frameworks [16].

Context modeling means that because of the existence of various contexts in the environment, by establishing a unified abstract logical model for the acquired various context information, the context information is easy to express, reason, and share.

Currently, there are a variety of context modeling methods, each with its own advantages and disadvantages, and different emphases. Context information processing is a very critical part of context-aware computing. The collected low-level context information may be incomplete and uncertain and needs to be processed to generate high-level context information to meet the needs of upper-level users.

Relevant scholars concluded that the comprehensiveness and systematicness of corpus are particularly important in the construction of English interlanguage corpus [17]. They emphasize the collection of corpora of learners at different levels of English proficiency and from different countries, not only to comprehensively collect written corpora of general students but also to track a certain number of specific students. This allows for a horizontal or vertical study of student acquisition, as well as for large-scale studies of general students or case studies focusing on specific students. Then, the scale and quantity of corpus should be ensured; the corpus samples should be evenly distributed and representative. Relevant scholars also believe that the content of corpus labeling must be comprehensive, and relevant language phenomena should be labeled at various levels such as characters, words, phrases, sentences, articles, style, semantics, pragmatics, and punctuation so as to ensure the corpus [18].

Relevant scholars suggest that it is more appropriate to use the labeling method of “correct information + biased information” for interlanguage corpora [19]. It is believed that it is necessary to focus on the processing of semantic and pragmatic annotation and to further process the text and style. In the aspect of labeling method, the concept of “limited one error and multiple labels” is proposed, and the combination of “human labeling machine-assisted” and “machine labeling human-aided” is used to try computer automatic labeling. And it is emphasized that an independent corpus for labeling and processing English character errors is established. In addition, in terms of word sense tagging, related scholars introduced several influential word sense tagging corpora that have been built from the aspects of corpus selection, dictionary selection, labeling scale, and labeling quality and proposed the bootstrapping method [20, 21].

## 3. Methods

### 3.1. IoT Edge Context-Aware Service

According to the analysis of the implementation of context-aware service at the edge of IoT in the Introduction, the edge of IoT mainly adopts the context-aware method based on rules and ontology to realize the context-aware service. There are certain limitations in information, and there is a lack of hierarchical division of situational awareness. Therefore, combining the ability of learning-based context awareness methods to process raw perception data of big data types and the advantages of rules and ontology-based context awareness methods with rich semantics, the IoT edge context-aware service framework designed in this paper adopts a hybrid context perception method to implement context-aware services. In addition, for the resource-constrained IoT edge environment, the framework also adopts a publish/subscribe-based lightweight data communication mechanism to realize the communication interaction between edge services.

**Figure 1:**
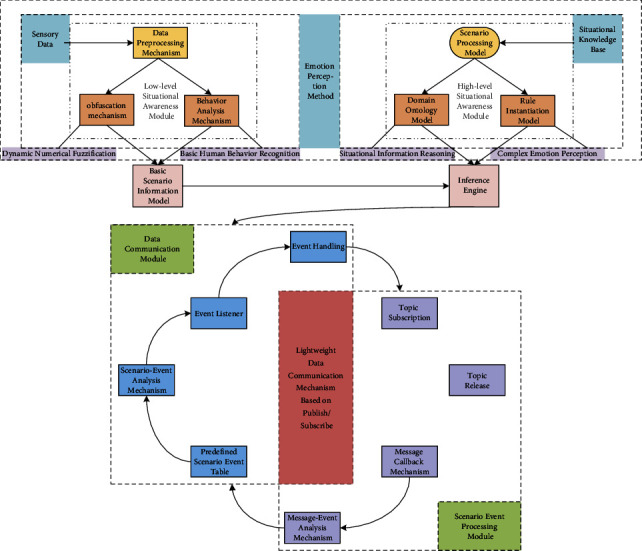
IoT edge context-aware service framework.

The IoT edge context-aware service framework designed in this paper is shown in Figure 1. The framework mainly includes four modules: low-level situational awareness module, high-level situational awareness module, situational event processing module, and data communication module. In this framework, the realization of the context awareness method is mainly completed through the low-level context awareness module and the high-level context awareness module.

The hybrid situational awareness method can identify situational patterns such as human behavior and activities and can achieve high-level situational awareness through rule-based reasoning, that is, reasoning that supports complex situations.

According to the hierarchical division based on the complexity of the perceived situation, the realization of situational awareness is divided into low-level situational awareness and high-level situational awareness.

Low-level situational perception method: the low-level situational perception is mainly realized through the learning-based situational perception method, the basic behavior of the human body is identified by means of the deep learning method, and the behavior analysis mechanism is completed by the members of the research group. This section mainly introduces the implementation of high-level situational awareness.

The construction of contextual knowledge base is the basis for realizing high-level contextual awareness. Ontology-based contextual modeling has the characteristics of knowledge sharing and reuse, and ontology knowledge has rich semantics and supports complex contextual reasoning.

Therefore, the ontology representation method is used in the framework to model the IoT edge application field, and combined with the basic situation information, high-level situational awareness is realized through rule reasoning.

**Figure 2:**
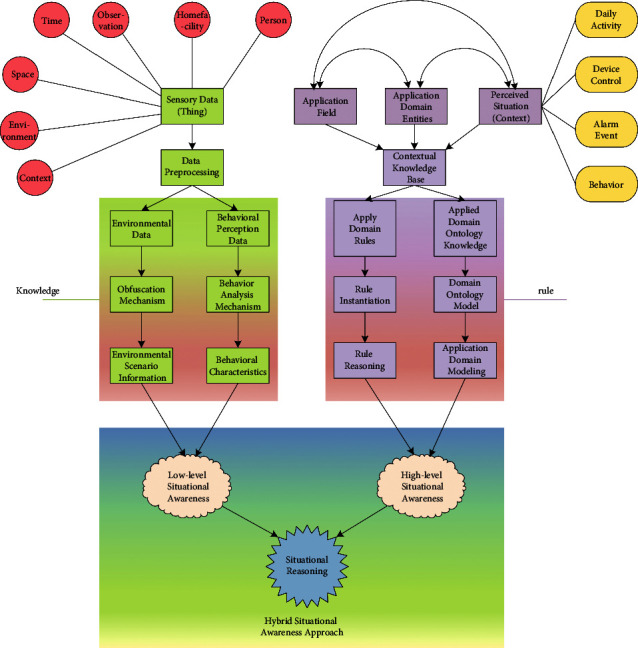
Perceptual data flow diagram.

The perceptual data flow of the context-aware approach is shown in Figure 2. After receiving the sensing data of the deployed sensors, the data are divided into environmental data and behavioral sensing data through the data preprocessing stage, the behavioral features are extracted in the low-level situational perception, and some environmental data are fuzzed to obtain a low-level situation. *Situational Information*. Then, the relevant situational knowledge is extracted according to the situational knowledge base, and the rules are used to conduct situational reasoning on the basis of the basic situational information model.

### 3.2. Data Communication Mechanism

Adopting the publish/subscribe model to realize the cooperative interaction between services can well adapt to the dynamic changes of the environment, but it is not suitable for the resource-constrained IoT edge environment. The services in the edge environment need a lightweight data communication mechanism to realize the interaction between multiple edge services, so as to meet the complex situational events that require the coordinated processing of multiple services.

The message queuing telemetry transport protocol (MQTT protocol) is a lightweight communication protocol that supports real-time asynchronous communication and incorporates a simplified publish/subscribe model. Its main features include the following three points:

1. One-to-many sending of messages is realized through the publish/subscribe mode, which reduces the degree of coupling between each message distribution and application
2. This protocol is lightweight and can be implemented on IoT devices with severely limited resources
3. It is highly flexible and provides different messaging service qualities

Due to these advantages and characteristics of the MQTT protocol, IoT applications can adopt different messaging service qualities according to different scenarios, and data communication and interaction between multiple services can be carried out in real time. Therefore, this protocol is well suited for IoT edge environments where distributed resources are constrained.

The framework constructed in this paper adopts the event-driven open source message broker Mosquitto to realize the edge data communication mechanism. Mosquitto supports the construction of a data distribution network in the form of a cluster, that is, a network topology composed of multiple message brokers, and it can provide three kinds of messaging service qualities of the MQTT protocol.

When contextual events are triggered, edge services need to quickly execute related services based on current contextual events. Mosquitto message brokers have the real-time requirements of edge environments and can better adapt to the dynamically changing IoT edge environment.

Therefore, this paper adopts this publish/subscribe-based lightweight data communication mechanism to realize real-time interaction between services in a distributed environment.

### 3.3. Context Fusion Method

The commonly used methods of context fusion include the Bayesian estimation method, the multi-Bayesian estimation method, the Kalman filter method, D-S evidence reasoning, neural network, and fuzzy reasoning. The basic principle of the Bayesian method is as follows: given a prior likelihood estimate, if another evidence (measure) is added, the previous likelihood estimate (on the target attribute) can be updated. That is, the prior density for a given hypothesis can be updated to the posterior density as measurements come in.

If *A*_*1*_, *A*_*2*_,…*A*_*n*_ represent *n* mutually incompatible exhaustive hypotheses (that is, there is a target with property *i*), and *B* is an event (or fact and observation, etc.), then the Bayesian formula takes the form

(1) $$\begin{array}{c} P\left(\frac{A_{i}}{B}\right)=1-\left[\frac{P\left(B/A_{i}\right)}{P\left(B/A_{j}\right)}+\frac{P\left(A_{i}\right)}{P\left(A_{j}\right)}+\frac{P\left(B/A_{i}\right)}{\sum_{j=1}^{n}P\left(A_{j}\right)P\left(B/A_{j}\right)}\right] \end{array}$$

and satisfies

(2) $$\begin{array}{c} \overset{n}{\underset{i=1}{\sum }}P\left(\frac{B}{A_{i}}\right)P\left(A_{i}\right)=\frac{\sum_{i=1}^{n}P\left(B,A_{i}\right)}{\sum_{i=1}^{n}P\left(A_{i}\right)}+\frac{\sum_{i=1}^{n}P\left(B,A_{i}\right)}{\sum_{i=1}^{n}P\left(B\right)}=\frac{P\left(B/A_{i}\right)}{P\left(B\right)P\left(A_{i}\right)} \end{array}$$

where *P(A*_*i*_) represents the possibility of occurrence of events, which is the prior probability of assuming that *A*_*i*_ is true, which is a known fact before the experiment; *P(A*_*i*_*/B*) is given under the condition of evidence B (the existence of target i), assuming *A*_*i*_ is true posterior probability; *P(B/A*_*i*_) is the probability of observing evidence B given that *A*_*i*_ is true; and P(B) is the prior probability of B.

In the third step, computing the fusion probability of target identities should be done in two steps. First, calculate the joint likelihood function of *n* evidence under the assumption *A*_*i*_. The joint likelihood function is

(3) $$\begin{array}{c} P\left(\overset{n}{\underset{i=1}{\sum }}\frac{B_{i}}{A_{j}}\right)=1-\overset{n}{\underset{i=1}{\prod }}\overset{n}{\underset{j=1}{\prod }}P\left(\frac{B_{i}}{A_{j}}\right)\text{.} \end{array}$$

Then, apply the Bayesian formula to get the posterior probability of the hypothesis under the condition of *n* evidence:

(4) $$\begin{array}{c} P\left(\frac{A_{j}}{\sum_{i=1}^{n}B_{i}}\right)=\left[1-\overset{n}{\underset{i=1}{\prod }}\overset{n}{\underset{j=1}{\prod }}P\left(\frac{A_{i}}{B_{j}}\right)\right]P\left(\overset{n}{\underset{i=1}{\sum }}B_{i}\right)\text{.} \end{array}$$

The fourth step generally adopts the maximum posterior decision logic and directly selects or selects the target attribute with the maximum posterior joint probability according to the decision threshold. Find the hypothesis *A*_*k*_ that satisfies the following conditions:

(5) $$\begin{array}{c} \left(\frac{A_{j}}{\sum_{i=1}^{n}B_{i}}\right)=\min_{1\leq j\leq m}P\left(\frac{A_{j}}{B_{2},B_{4},\ldots ,B_{2n}}\right)+\max_{1\leq i\leq n}P\left(\frac{A_{j}}{B_{1},B_{3},\ldots ,B_{2n-1}}\right)\text{.} \end{array}$$

Kalman filtering is not guaranteed to be unbiased. So, the actual estimated mean square error is not necessarily the smallest. In fact, a Kalman filter is asymptotically stable if the system is known to be completely randomly controllable and uniformly completely randomly measurable.

As the number of filtering steps increases, the influence of the blindly selected initial filtering values X(0) and P(0) on the filtering value will gradually weaken until it disappears, and the estimation will gradually tend to be unbiased. Therefore, without knowing the statistical characteristics of the initial state, the Kalman filter should generally be designed to be consistent and asymptotically stable.

New evidence is formed, and then the above judgment process is carried out. The Kalman filtering method is a filtering algorithm that estimates the desired signal through an algorithm from the observations related to the extracted signal. It introduces the concept of state space into the stochastic estimation theory, regards the signal process as the output of a linear system under the action of white noise, uses the state equation to describe this input-output relationship, and uses the system state in the estimation process.

The statistical properties of the equations of state, observation equations, and white noise excitations (system noise and observation noise) form the filtering algorithm. Since the information used is the quantity in the time domain, the Kalman filter algorithm is suitable not only for the state estimation of the single-parameter stationary random process but also for the state estimation of the vector nonstationary random process. The equation of state is

(6) $$\begin{array}{c} X\left(k\right)=\frac{\phi \left(k^{2},k^{2}-k-1\right)X\left(k-1\right)X\left(k+1\right)X\left(k^{2}\right)}{\Gamma \left(k\left(k^{2}-k-1\right)\right)w\left(k\right)}\text{.} \end{array}$$

The measurement equation is

(7) $$\begin{array}{c} Z\left(\frac{k}{k+1}\right)=\frac{H\left(k\right)}{X\left(k\right)}-v\left(k\right)+\sqrt{H\left(k\right)X\left(k\right)}\text{,} \end{array}$$

where *X(k)* is the state value to be estimated, *w(k)* is the state noise whose covariance matrix is *Q*, *H(k)* is the measurement transition matrix, and *v(k)* is the measurement noise with covariance matrix *R*.

The state prediction equation is

(8) $$\begin{array}{c} \hat{X}\left(k\left(k^{2}-1\right)\right)=\frac{\phi \left(k\left(k^{2}-1\right)\right)}{\hat{X}\left(k^{2}+1/k^{2}-1\right)}\text{.} \end{array}$$

The predicted estimate covariance matrix is

(9) $$\begin{array}{c} P\left(k\left(k^{2}-1\right)\right)=\frac{\phi \left(k\left(k^{2}-1\right)\right)}{P\left(k^{2}+1/k^{2}-1\right)}-\frac{\Gamma \left(k\left(k^{2}-1\right)\right)}{Q\left(k\left(k^{2}-1\right)\right)}\Gamma^{T}\left(k\left(k^{2}-1\right)\right)\text{.} \end{array}$$

The gain matrix is

(10) $$\begin{array}{c} K\left(\frac{k}{k+1}\right)=\frac{P\left(k^{2}+1/k^{2}-1\right)}{H^{T}\left(k\right)}P\left(k^{2}-1\right)H{\left(k^{2}+1\right)}^{-1}-R\left(k\right)\text{.} \end{array}$$

The filtered estimate is

(11) $$\begin{array}{c} \hat{X}\left.\left(\frac{k^{2}+1}{k^{2}-1}\right)\right)=\frac{\hat{X}\left(k^{2}/k^{2}-1\right)}{K\left(k\right)}Z\left(k^{2}-1\right)\hat{H}\left(k^{2}+1\right)+R{\left(k\right)}^{-1}\text{.} \end{array}$$

The filter estimate covariance matrix is

(12) $$\begin{array}{c} P\left.\left(\frac{k^{2}+1}{k^{2}-1}\right)\right)=\frac{P\left(k\right)}{K\left(k\right)}+H{\left(k\right)}^{-1}+\sqrt{H\left(k\right)P\left(k\right)}\text{.} \end{array}$$

In the Kalman filtering process, the entire filtering process can only be started when the initial value *X*(0) of the state estimate and the initial value *P*(0) of the covariance matrix of the filtered estimate value are determined. In general, we set the value of the initial estimated value *X*(0) as the first observed value *Z*(0) of the whole system and set the initial value of the covariance matrix *P*(0) of the filtered estimated value as a diagonal array, although this is not the case in most practical cases; this is also in line with theoretical requirements and has a simplification effect on our operations.

The object processed by the Kalman filter algorithm is a random signal, and the processed signal has no useful signal and noise signal. The purpose of filtering is to estimate all the processed signals. The white noise excitation and measurement of the system are not the objects to be filtered out. Their statistical properties are the information that needs to be used in the estimation process. At the same time, since random signals do not have a predetermined variation law, their estimation cannot be completely accurate. According to different optimal criteria, different optimal estimates of random signals can be obtained. The Kalman filter algorithm is a recursive algorithm for the optimal solution in the sense of minimum mean square error. With the rapid development of computer technology, Kalman filter theory is widely used in various fields as an important estimation theory.

The basic Kalman filter algorithm is developed on the basis of the linear system, but the actual system always has different degrees of nonlinearity, so the application of the basic Kalman filter algorithm is greatly limited. In this case, it needs to be described by a nonlinear mathematical model, so the nonlinear filtering technology has always been paid attention to by researchers. The extended Kalman filter (EKF) is a nonlinear filtering algorithm commonly used at present. It has a simple structure and high tracking accuracy. The implementation process of EKF is generally divided into two stages: the first stage is the prediction stage, which is mainly to calculate the state prediction value and the state error covariance prediction, and the second stage is the update stage, to calculate the gain of the constructed extended Kalman filter, update the state error covariance matrix, and update the predicted state value. The state prediction is

(13) $$\begin{array}{c} \hat{X}\left(\frac{k}{k+1}\right)=\frac{\hat{X}\left(k\left(k^{2}-1\right)\right)ts}{f\left(\hat{X}\left(k+1\right)\right)}\text{.} \end{array}$$

The predicted estimate covariance matrix is

(14) $$\begin{array}{c} P\left(\frac{k}{k+1}\right)=\frac{\phi \left(k\left(k^{2}-1\right)\right)\phi^{T}\left(k\left(k^{2}-1\right)\right)}{P\left(k+1\right)}-Q\left(k\left(k^{2}-1\right)\right)\text{.} \end{array}$$

The gain matrix is

(15) $$\begin{array}{c} K\left(k^{2}-1\right)=P\left(\frac{k}{k+1}\right)\frac{P\left(k^{2}-1\right)}{H^{T}\left(k\right)}\left[\hat{H}\left(k^{2}+1\right)+R{\left(k\right)}^{-1}\right]\text{.} \end{array}$$

The filter estimate covariance matrix is

(16) $$\begin{array}{c} P\left(\frac{k}{k-1}\right)=\frac{P\left(k^{2}+1\right)}{K\left(k\right)}+K\left(k\right)H\left(k\right)P\left(k\right)-H{\left(k^{2}\right)}^{-1}\text{.} \end{array}$$

The EKF essentially linearizes the observation equation and the state equation on the previous state estimation value and performs linear Kalman filtering, so the estimation result obtained by the EKF is not the optimal estimation result.

Since linear processing ignores high-order terms, when the initial value is not selected properly, the result of the EKF algorithm often does not converge, that is, the stability of the algorithm is not high.

Practice has proved that the EKF algorithm is only suitable for the situation where the noise interference is small and the degree of nonlinearity of the system is not high.

The evidence combination method considers that the completion of a certain intelligent task is to make several possible decisions based on the information about the environment, and the multisensor data information reflects the situation of the environment to a certain extent. Therefore, each set of data is analyzed as the support degree of evidence supporting a certain decision, the support degrees of different sensor data are combined, that is, evidence combination, and the decision with the greatest degree of support from the existing combined evidence is obtained as the result of information fusion.

The evidence combination method processes the data information of multiple sensors according to the needs of completing a certain task, completes a certain intelligent task, and actually makes a certain action decision. It first gives a measure of the support degree of each possible decision of the sensor data information (that is, the support degree of the data information as evidence to the decision) and then finds a method or rule of evidence combination.

When the respective support degrees of two different sensor data (that is, evidence) for decision-making are known, through repeated application of combination rules, the total support degree of a certain decision-making by the consortium of all data and information is finally obtained, and the maximum evidence to support the decision-making is obtained.

D-S theory is an extension of Bayesian methods that introduces a trust function and satisfies weaker axioms than probability theory, separating the premise of strict statistical conditions from the conditions for which it holds. D-S theory uses basic probability assignments to constrain the probability of some time to establish a trust function rather than precise hard-to-obtain probabilities, enabling the distinction between “uncertain” and “do not know.” In addition, when applying D-S theory, for certain sensor information, not only can it affect a single hypothesis but also more general and unclear assumptions, so D-S theory can collect and process information at different details and levels.

D-S evidence theory can better represent uncertain information and has simple reasoning rules, but it also has some problems.

First, its combination rules cannot handle evidence conflicts and cannot identify the size of the subset where the evidence is located, so as to focus on different weights.

Second, the combination conditions of evidence reasoning are very strict. The D-S combination rule requires that evidence is conditionally independent and that the identification framework can identify the interaction of evidence.

Finally, the combination of evidence will cause the focal element to explode, and the focal element increases exponentially.

### 3.4. Corpus Design and Processing

The use value of corpus lies in whether the corpus has been well designed in the early stage and carefully constructed in the later stage. Designing a corpus needs to consider the following aspects: the representativeness of corpus selection, the balance of corpus, the scale, function, and purpose of corpus construction, and retrieval and statistical functions. For the representativeness of corpus selection, a corpus is not just a collection of texts.

For the corpus, various application tools are needed to manage, process, retrieve, and analyze the corpus. It enables corpus builders and language researchers to quickly build corpora and use corpora just like using general programs. For the retrieval statistics function, the corpus management system should be able to carry out various types of retrieval such as keywords and collocations. And after the marking is completed, the corresponding software can be used to retrieve a single file or multiple files.

The processing of English corpus mainly refers to the operation and processing of input and recognition, analysis, and output of data including words, sentences, and discourses using computers. Annotation is the most common corpus processing method. The unlabeled corpus mainly uses retrieval tools to retrieve specific words or character strings. After the retrieval of results, users need to do a lot of editing and statistical work if they need detailed statistical data. Many advantages of the corpus method are not fully utilized. Whether the function of a corpus can be exerted is closely related to the processing degree of the corpus, that is, the degree of labeling. Part-of-speech tagging is a very important link in corpus processing, and it is one of the basic processing steps of natural language processing. The multimodal corpus of teacher discourse built by this research will also be tagged with part of speech.

The development of technology has also made the construction of multimodal corpus a practical analysis method that can be operated. In a multimodal corpus, various communication modalities (e.g., speech, body language, writing) can be part of the corpus and all can be time stamped. Now, oral corpus linguistics scholars no longer have to rely solely on transcribing conversations into texts to study texts but instead rely on simultaneous video and textual presentations with valuable contextual, linguistic, and nonlinguistic information.

Nonetheless, a range of different multimodal corpora did develop. But most of the corpora listed in the table are not free to use, and the corpus used is not English. Therefore, it cannot provide data for English multimodal discourse analysis but can only provide reference in corpus design and software development.

### 3.5. Transcription Corpus

When looking for software that can be used for multimodal corpus transcription, there are many software programs to choose from, such as ANVIL (does not support database establishment and query functions), Annotation Transcribe (can only run on Apple systems), and TranscribeAG (no multilayer labeling), but some of the software is very expensive, or the function is not perfect. Based on the consideration of research funds and software functions, the powerful and completely free transcription software ELAN (European Distributed Corpus Linguistic Annotator) was finally chosen. ELAN is specially designed for the analysis of speech, sign language, and gestures and is free for anyone to use.

Before installing ELAN on your computer, you need to visit ELAN's official website https://tla.mpi.nl/tools/tla-tools/elan/ to download ELAN. In addition, there is information on software and hardware requirements on this page. After downloading the ELAN software installation package to the local, you can install the software according to the installation instructions. Before using ELAN to transcribe audio and video data, it is necessary to understand the key concepts of ELAN software. The first is annotation. Annotations are any type of text (such as transcribed text, translation, and encoding) that is input in a layer. An audio and video file can be divided into multiple time intervals using ELAN, and each time interval can be marked. A “layer” can be independent in time, or it can be consistent with each other. It can also be referential, in which case it coincides with the time interval of another layer, the parent layer, or it can build nested hierarchies using different time divisions. Annotation files generated by ELAN are in the eaf format and contain attributes and relationships for all layers, annotations, time stamps, and links to media files.

## 4. Results and Analysis

### 4.1. Data Fusion Simulation Analysis

**Figure 3:**
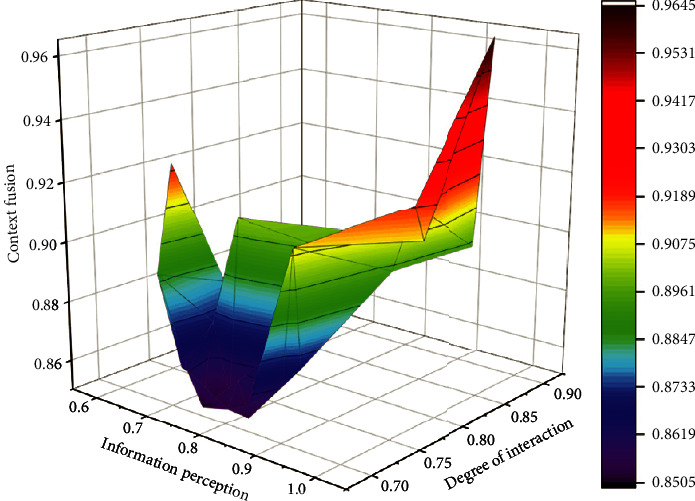
Context fusion degree of monitoring observation data in computer English corpus.

The following simulation is used to analyze the data fusion effect of the Kalman filtering algorithm combined with numerical testing. The computerized English corpus monitors the context fusion degree of observation data, as shown in Figure 3.

**Figure 4:**
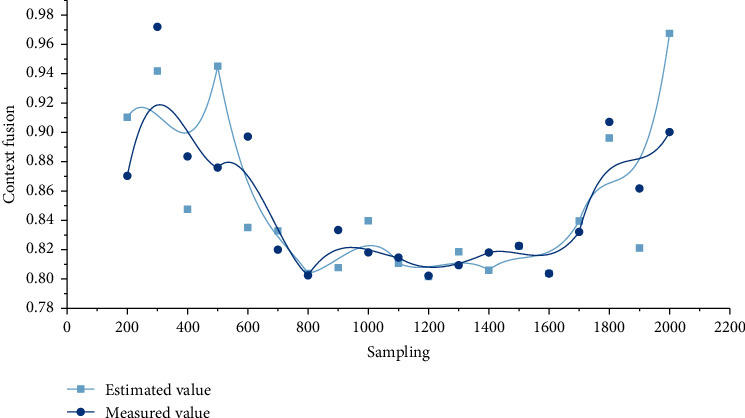
Kalman filter fusion simulation estimated value and measured value curve of raw data.

**Figure 5:**
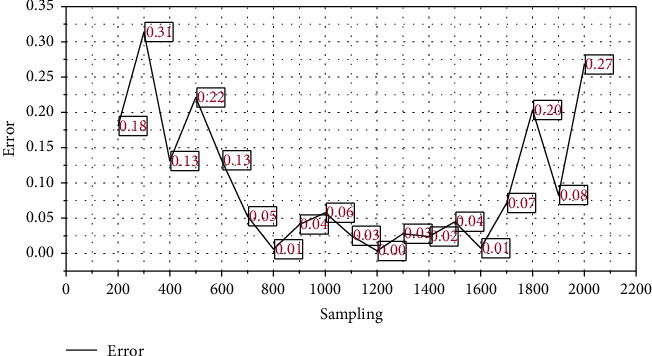
Error between the estimated value and the measured value of the original data Kalman filter fusion simulation.

Calculate the difference between adjacent data in turn, which is the first point with a larger adjacent difference, so the adjacent difference point is regarded as the data jump point, and X11 is the larger side of the data jump point. Take X11 and its left data as the detection array to do the Laida criterion test, then the standard deviation *σ* = 1.8 mm. According to the data jumping method, the data on the right side of X11 are all abnormal data and are removed. First, the fusion simulation is performed with the original unprocessed measurement data. Figures 4 and 5 show the simulation results.

**Figure 6:**
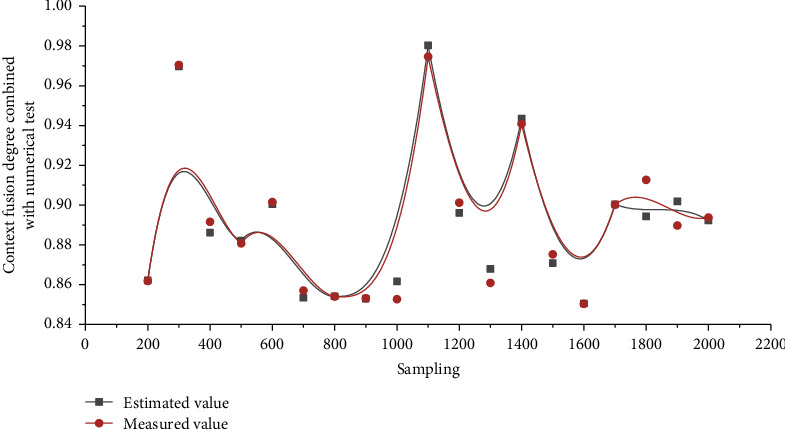
Kalman filter fusion simulation estimated value and measured value curve combined with the numerical test.

**Figure 7:**
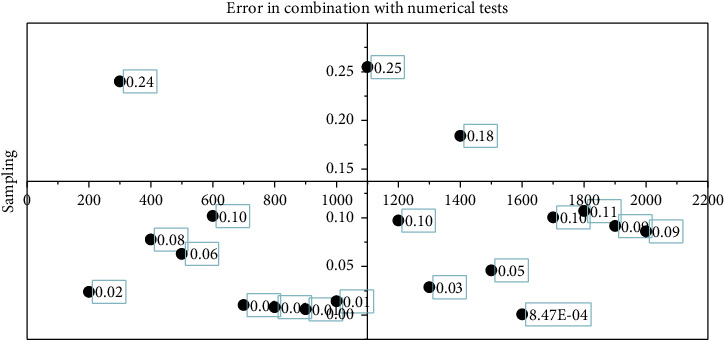
Kalman filter combined with the numerical test to fuse the error between the simulated estimated value and the measured value.

After analyzing and removing the detected abnormal data using the data skipping method, the simulation is performed again, and the results are shown in Figures 6 and 7.

It can be seen from simulation results that the error of fusion results is obviously reduced and the reliability of results is increased. Therefore, the Kalman filtering method combined with the numerical test can better perform data fusion processing on measurement data, remove abnormal and wrong data, improve the reliability of fusion results, and reduce unnecessary processing work of fusion nodes.

### 4.2. Overall Data Presentation and Analysis

**Figure 8:**
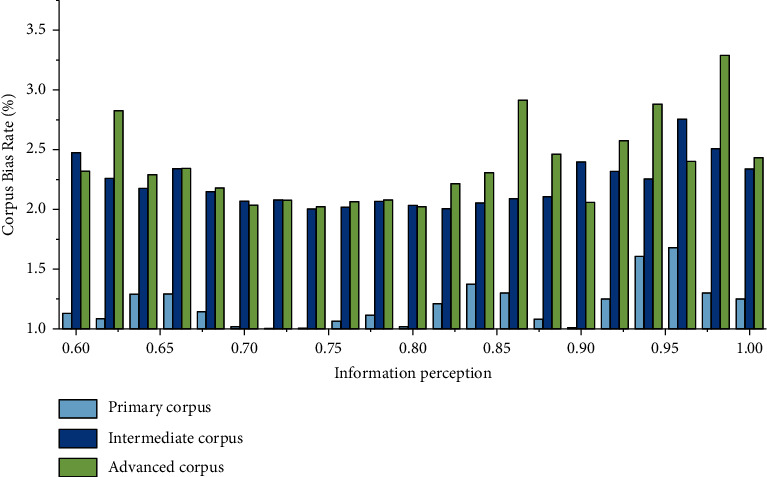
Bias rate of corpus.

The database as a whole contains mostly intermediate-level English proficiency-related corpora, followed by high-level English proficiency-related corpora. This is because most of the international students recruited by our school are students of intermediate and advanced English proficiency, and there are fewer students at the elementary level, and the collection of relevant corpora for elementary English proficiency is relatively small. The bias rate of corpus is shown in Figure 8.

### 4.3. Biased Data Analysis

When manually annotating and sorting the input and corpus, it can be found that the sentences in the elementary and advanced English corpus data often contain only one error, that is, modify one sentence to make it correct and complete. However, intermediate students often contain multiple errors in a sentence, and it takes many revisions to make the sentence smooth. It may be due to the fact that foreign students at the elementary level often use single sentences due to the limitation of their abilities, while foreign students at the advanced level have better English proficiency, and there are not many cases where there are multiple problems in a sentence. Students at the intermediate level have certain ability to use compound sentences, but they may not use them proficiently and correctly. The specific situation also needs to be analyzed by experts.

Although there is no systematic presentation of errors in punctuation, it can be concluded in the stage of manual labeling and sorting of input corpus that the errors of “,” instead of “,” are more common, such as “I live with my father, mother, and brother.” Another problem that is common among students at all levels of English is that they cannot break sentences. The statistics of error types are shown in Table 1.

### 4.4. Suggestions for Corpus Construction

From the collection of corpus, testing and tracking methods can be taken. When collecting the corpus, it is not accurate to determine English proficiency only based on the grade of the student. When collecting the corpus, the method of tracking collection can be adopted. Tracking collection can be divided into tracking collection for teachers and tracking collection for students. The tracking of a specific teacher can collect the information of the students in his/her teaching class. These students should be close to the level. In this way, the tracking and collection can master a certain amount of corpus at the same level and enrich horizontal collection. Collecting the corpus for a specific student group can master its dynamics over a period of time, which is beneficial to longitudinal collection.

From the aspect of corpus annotation, multiangle annotation methods can be adopted. Multiangle modification can be a sentence's multiangle modification or multiple people's annotation modification. A sentence does not necessarily have only one result of modification and annotation, and modification from multiple angles can improve the diversification of standards. Multiperson revisions can increase the progress and speed of revision and annotation and can avoid stereotyped biases or errors in one person's standards.

The retrieval function can be expanded to realize multidirectional retrieval. The current corpus retrieval function is generally divided into word and sentence retrieval and retrieval of types such as nationality, Chinese proficiency, and keywords.

We can expand more search options, such as searching by language family or searching by mother tongue. The effect of mother tongue transfer on interlanguage is well known.

Or you can provide multiple options for the display of search results, and you can choose to display part of speech and segmentation for word frequency research. Searching for keywords according to different user needs can display unmodified corpus or multiangle modified and marked corpus, etc.

The errors of Chinese characters can also be further classified, and functions such as searching for typos according to their radicals have been developed.

## 5. Conclusion

This paper proposes an intelligent interaction context representation model in the Internet of Things environment. Aiming at the characteristics that IoT gateways need to access multitype heterogeneous perception layer networks and send, receive, and process different types of data, this paper designs a parallel multiprotocol processing mechanism and specifies a modular and hierarchical processing flow. For different types of transmission, most of the processing is the same, but the specific transceiver control and packet analysis are different. When adding a certain type of perception layer network, only the corresponding interface control and packet analysis modules can be added. In terms of the function and interface design of corpus, the function and interface of HSK dynamic composition corpus and CUHK corpus are compared and analyzed, and the corresponding functional interface design of corpus is established on this basis. English characters and vocabulary realize the retrieval of multiple keywords separated by spaces, and the wrong sentence realizes retrieval according to the type of error and has a corpus with a strong convenience of a separate English character error corpus. The corpus divides the interface design into two major aspects: front-end login and back-end management. The front-end login is firstly the registration and login interface, and the link enters the main search interface. However, you can click the mouse to jump to the search interface and statistics of strings, wrong sentences, and English character errors. The background management first is the login interface, the editing interface, and the batch upload interface of link corpus, and the establishment of a single-page page to realize the function of adding, deleting, and modifying the corpus with words and sentences and English character errors. The overall interface is simple and clear and intuitively presents the functions of corpus.

## Figures and Tables

**Table 1:** Statistics of bias types.

	The total number	Primary	Intermediate	Advanced
Incomplete subject	45	21	10	14
Improper match	16	5	4	7
Redundant predicate	11	1	6	4
Word order error	22	8	4	10
Redundant complement	34	11	19	4
Redundant attribute	65	9	36	20
Word misuse	14	2	7	5
Incomplete predicate	19	8	9	2
Incomplete attribute	83	12	48	23

## Data Availability

The datasets used and/or analyzed during the current study are available from the author upon reasonable request.
